# BODY COMPOSITION CHANGE AND PHYSICAL FUNCTION AFTER INTENSIVE BEHAVIORAL WEIGHT LOSS INTERVENTION

**DOI:** 10.1093/geroni/igae098.3463

**Published:** 2024-12-31

**Authors:** Kacey Chae, Amie Bettencourt, Denise Houston, Eleanor Simonsick, Luigi Ferrucci, Rita Kalyani, Jeanne Clark, Kimberly Gudzune

**Affiliations:** Johns Hopkins University School of Medicine, Baltimore, Maryland, United States; Johns Hopkins University School of Medicine, Baltimore, Maryland, United States; Wake Forest University School of Medicine, Winston Salem, North Carolina, United States; National Institute on Aging, Baltimore City 0400 Baltimore City, Maryland, United States; National Institute on Aging, Baltimore, Maryland, United States; Johns Hopkins University School of Medicine, Baltimore, Maryland, United States; Johns Hopkins University School of Medicine, Baltimore, Maryland, United States; Johns Hopkins University School of Medicine, Baltimore, Maryland, United States

## Abstract

The LookAHEAD trial, an RCT of adults with obesity and diabetes, has demonstrated weight loss (WL) efficacy through intensive lifestyle intervention (ILI), but also a decline in lean mass (LM) after ILI. One potential concern with LM reduction is worsening physical function (PF), particularly among older adults. Our study objective was to evaluate the change in PF across different categories of body composition changes during ILI. We conducted a secondary analysis within the LookAHEAD trial, among ILI participants with dual-energy X-ray absorptiometry (DXA). The independent variables were 1). percent WL, 2). percent FM change, and 3). percent appendicular lean mass (ALM) change at 12 months (12M). The dependent variable was the change in self-reported PF at 12M, measured by the SF-36 (higher scores indicating better PF). At baseline, 628 participants (63% women) had mean age 58.3 years (SD 6.7), weight 96.1 kg (16.8), FM 39.8 kg (10.7), ALM 22.9 kg (5.1), and PF score 81.5 (17.6). Mean WL was 9.2% (6.3); PF scores increased in the WL ≥10% and 5-9.9% groups. Mean change in FM was -14.8% (11.7); PF scores increased in the high FM-loss, moderate FM-loss, and low FM-loss groups [high-loss vs. low-loss (p=0.02); high-loss vs. gain (p=0.04)]. Mean change in ALM was -4.8% (4.9); PF score increased in the high ALM-loss, moderate ALM-loss, and low ALM-loss groups, but the between-group differences were not significant (p=0.06). Results suggest that LM loss does not necessarily contribute to PF declines; FM loss may be a greater contributor to PF improvement.

